# Assessing Uncertainty in Spatial Exposure Models for Air Pollution Health Effects Assessment

**DOI:** 10.1289/ehp.9849

**Published:** 2007-05-10

**Authors:** John Molitor, Michael Jerrett, Chih-Chieh Chang, Nuoo-Ting Molitor, Jim Gauderman, Kiros Berhane, Rob McConnell, Fred Lurmann, Jun Wu, Arthur Winer, Duncan Thomas

**Affiliations:** 1 Department of Epidemiology and Public Health, Imperial College London; 2 Division of Environmental Health Sciences, School of Public Health, University of California, Berkeley, California, USA; 3 Department of Preventive Medicine, University of Southern California, Los Angeles, California, USA; 4 Sonoma Technology, Incorporated, Petaluma, California, USA; 5 Division of Epidemiology, School of Medicine, University of California, Irvine, California, USA; 6 School of Public Health, University of California, Los Angeles, California, USA

**Keywords:** air pollution, Bayesian analysis, lung function, measurement error, spatial exposure models

## Abstract

**Background:**

Although numerous epidemiologic studies now use models of intraurban exposure, there has been little systematic evaluation of the performance of different models.

**Objectives:**

In this present article we proposed a modeling framework for assessing exposure model performance and the role of spatial autocorrelation in the estimation of health effects.

**Methods:**

We obtained data from an exposure measurement substudy of subjects from the Southern California Children’s Health Study. We examined how the addition of spatial correlations to a previously described unified exposure and health outcome modeling framework affects estimates of exposure–response relationships using the substudy data. The methods proposed build upon the previous work, which developed measurement–error techniques to estimate long-term nitrogen dioxide exposure and its effect on lung function in children. In this present article, we further develop these methods by introducing between- and within-community spatial autocorrelation error terms to evaluate effects of air pollution on forced vital capacity. The analytical methods developed are set in a Bayesian framework where multistage models are fitted jointly, properly incorporating parameter estimation uncertainty at all levels of the modeling process.

**Results:**

Results suggest that the inclusion of residual spatial error terms improves the prediction of adverse health effects. These findings also demonstrate how residual spatial error may be used as a diagnostic for comparing exposure model performance.

Leading researchers have identified the development of models for assessing air pollution exposure within cities as a priority for future research (Brauer et al. 2003; Brunekreef and Holgate 2002; National Research Council 2002). In the present article we compare and evaluate four spatial models for assigning air pollution exposure at the within-community or intraurban scale. We assess how each model predicts exposure and affects health risks in the context of the Southern California Children’s Health Study (CHS; Peters et al. 1999a, 1999b). The CHS study assessed childhood lung function in 12 communities selected to represent a range of exposures. Effects of a correlated group of pollutants, including particulate exposure and nitrogen dioxide were associated with deficits in forced vital capacity (FVC, a measurement of lung volume) and forced expiratory volume in 1 sec (FEV_1_, a measurement of flow rate) (Gauderman et al. 2004, 2007; Molitor et al. 2006; Peters et al. 1999b). The data allow us to examine the effect of incorporating spatial residual errors into the modeling framework of Molitor et al. (2006), potentially explaining a spatial structure not accounted for by the exposure predictors. Therefore, the data serve as a foundation on which to test different exposure models with and without spatially distributed errors and to examine the role of exposure measurement error in air pollution studies.

Interest in assessing exposure at the intraurban scale has grown for a variety of reasons, including early evidence of the large adverse health effects that may emerge from this scale of analysis. For example, Hoek et al. (2002) reported a near doubling of cardiopulmonary mortality [relative risk = 1.95; 95% confidence interval (CI), 1.09–3.52] for Dutch subjects living near major roads in a cohort of 5,000 people, after control of many confounding variables. Although these findings may be robust, the basic exposure models used in these analyses may misclassify exposure because they treat the continuous air pollution field as a discrete entity, that is, either within or outside a specified distance from a road (Jerrett et al. 2005a, 2005b). Thus, questions remain about the validity of results from health effects studies that use exposure surrogates such as road buffers.

Other factors have heightened interest in assessing the relation between air pollution and adverse health effects at the intraurban scale. Empirical exposure studies have shown that for some pollutants associated with traffic, such as NO_2_ and ultrafine particles, variation within cities may exceed variations among central monitoring locations in different cities. Earlier studies from the United Kingdom indicate 2- to 3-fold differences in NO_2_ within distances of ≤ 50 m of a major road (Hewitt 1991), whereas U.S. studies suggest ultrafine particle concentrations are higher than background until about 300 m from highways during daytime hours (Zhu et al. 2002). The preliminary evidence of large health effects at the intraurban scale and the empirical findings that air pollution exposure varies more within than between communities imply that the most meaningful exposure gradient for research on the adverse health effects of air pollution may occur at the intraurban scale.

Assessing pollution distributions at the intraurban scale has proved challenging because of the lack of routinely collected data, but a new class of models (Jerrett et al. 2005a) that uses geographic information systems (GIS) to integrate existing information now shows promise. These models combine available data on monitoring concentrations, land use, meteorology, time–activity patterns, and emissions. Calibrated exposure models based on this information can identify variation in air pollution concentrations within small areas. Resulting pollution surfaces can then be overlaid on georeferenced study data to assign exposure to individuals at their place of residence, work, or some combination of these microenvironments.

There is little doubt that air pollution levels are spatially autocorrelated within cities, and it is also possible that residual health outcomes would be autocorrelated, either because of imperfect estimates of air pollution levels or because of other unmeasured risk factors not represented in the prediction. This implies that standard regression methods for exposure assessment that assume independence are not valid and would be expected to yield biased variance of parameter estimates and inefficient significance tests. Furthermore, one would expect that methods that exploit these spatial correlations should lead to better prediction of individual exposures by “borrowing strength” from measurements at neighboring locations and improving the imputation of exposures for individuals for whom no measurements are available. To date, few models have exploited spatial dependence to refine estimates of air pollution exposure within cities or the associated prediction of health outcomes.

In the present article we build on epidemiologic, land use, air pollution, and emission data to produce estimates of long-term NO_2_ exposure for 11 CHS communities. These estimates will be integrated within a Bayesian statistical framework to assess *a*) the marginal benefit of moving from less to more refined exposure models, *b*) the specific contribution of spatial terms to reducing exposure error, and *c*) the role of uncertainty in health effects analysis.

## Materials and Methods

**Figure 1:**
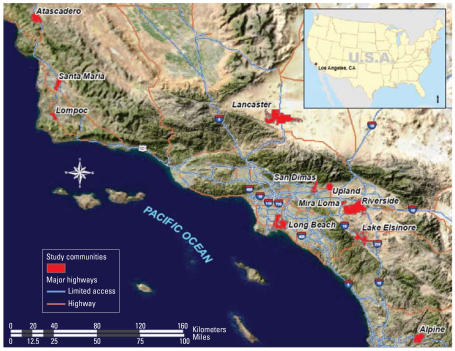
Location map of communities in CHS study. All communities are located in Southern California (see inset)

We obtained data used in this study from the Southern California CHS, a study of over 5,000 children enrolled from schools in communities selected to represent the range and mix of regional ambient air pollution (Peters et al. 1999b). We obtained resident-level pollution data from a study conducted in 2000 (Gauderman et al. 2005), in which out-door NO_2_ concentrations were measured at 233 homes of CHS children selected from 11 of the 12 communities (Figure 1; the mountain community of Lake Arrowhead was excluded because the home addresses could not be accurately geocoded). Subjects were selected randomly from within two strata defined by the distributions of local traffic counts within each community. Two-week average measurements of NO_2_ concentrations were taken in 2000 at each home, one in summer and one in winter. Subjects’ home and school addresses were geocoded for exposure assignment and specification of the spatial correlation structure, as described below. The predicted average NO_2_ exposure from the California line source dispersion (CALINE4) model (Benson 1989) and distance from the residence to the nearest freeway were also selected as standard exposure models. Details of the sampling and measurement protocols can be found in Gauderman et al. (2005) and of the specification of the exposure prediction variables in Molitor et al. (2006).

The household pollution data that we analyzed are from a study conducted in 2000, in which outdoor NO_2_ concentrations were measured at 233 homes of CHS children during one 2-week period in the summer and one 2-week period in the winter. Subjects were approximately 10 years of age at enrollment and between 14–17 years of age when the NO_2_ measurements were taken. Here, we focus on the relationship between exposure to NO_2_ and FVC, a standard spirometric measure of lung volume (Gauderman et al. 2004), which allowed for direct comparison with previous analyses (Molitor et al. 2006). Previous studies have linked local traffic and regional air pollutants to this outcome (Ackermann-Liebrich et al. 1997; Gauderman et al. 2007). Lung testing maneuvers were performed using a standardized protocol based on American Thoracic Society recommendations, modified for children (Peters et al. 1999a).

In the present article we extend the approaches used by Gauderman et al. (2005) and Molitor et al. (2006) by including extra spatial residual terms. This addition is potentially beneficial because subjects living in the same town might exhibit geographic cluster effects of NO_2_ exposure or some other unmeasured covariate. We tested this cluster effect by including the spatial variance component in the model similar to Borgoni and Billari (2003). To extract the unobserved spatial error, the spatial patterns of subjects were specified through the use of explicit spatial connectivity matrices for subjects in different towns and those within the same town. The formulation of the spatial models is explained below.

### Model

**Figure 2:**
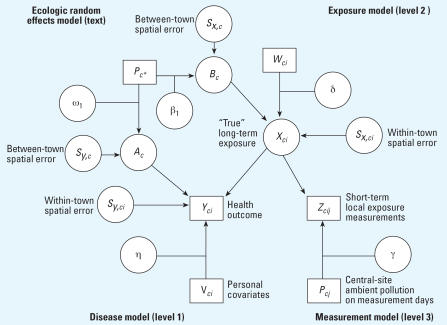
Directed acyclic graph (DAG) for entire model.

Similar to recent studies (Chaix et al. 2006), NO_2_ serves as a proxy to local traffic pollution exposure in our model. In our previous study (Molitor et al. 2006), the unified Bayesian framework for the multilevel analysis improved the estimates of the effect of NO_2_ exposure on lung function in children with incomplete outcome measures by fitting the multilevel models as a unit. In this present article, we extend this framework to include spatial autoregressive error terms, and we compare the estimates of NO_2_ exposure obtained from these models that include the spatial error terms with models that specify only independent errors. First, we define the following notations for the subject *i* in town *c* in season *j: a*) *Y_ci_* denotes measurements of lung function (FVC); *b*) *Z_cij_* denotes observed subject-level outdoor NO_2_ exposure measurements; *c*) *X_ci_* denotes the “true” unobserved annual outdoor household-level NO_2_ exposure level; *d*) *P_cj_* denotes season-specific central-site exposure; *e*) *W_ci_* denotes a vector of household-level NO_2_ exposure predictors, including distance to the nearest major road, categorized as distance to the nearest freeway based on the road buffer (> 300 m; 150–300 m; 75–150 m; < 75 m), traffic density within 150 m of subjects’ locations, and predicted NO_2_ concentration from the CALINE4 model; *f* ) *V_ci_* is a vector of personal covariates that affect the lung function, specifically including age, sex, race/ethnicity, height, body mass index (BMI), cohort enrollment group, height, exercise, smoking behavior, asthma, and respiratory illness at the time of lung function measurements; *g*) *A_c_* and *B_c_* are the community-specific intercepts in the lung function and exposure models, respectively; *h*) *s_y,ci_* and *s_X,ci_* are in turn the within-community spatial errors for the lung function and the long-term NO_2_ exposure. All NO_2_ levels, both observed and unobserved, are on the log scale. This analytical framework consists of the following three-level hierarchical models, lung function (level 1), exposure (level 2), and measurement (level 3) models, respectively:


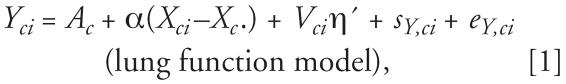



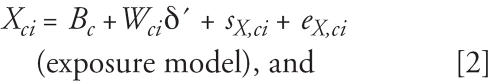



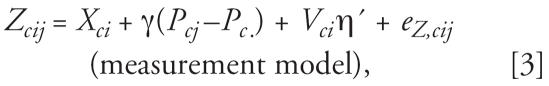


where *X_c._* and *P_c._* are community-specific averages of *X_ci_* and *P_cj_*. The community-specific intercepts *A_c_* and *B_c_* were further modeled as:


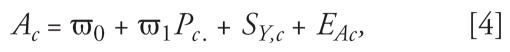


and


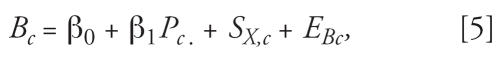


where *S_Y,c_* and *S_X,c_* are between-community spatial errors for Equations 4 and 5, respectively. In addition, the terms *e_Y,ci_* , *e_X,ci_*, *e_Z,ci_* , *E_Ac_*, and *E_Bc_* are assumed to be normally distributed random errors with zero means and variances σ*_Y_* ^2^, σ*_X_* ^2^, σ*_Z_* ^2^, σ*_h_*^2^, and σ*_k_* ^2^, respectively. All the spatial error terms, *s_Y,ci_* , *s_X,ci_* , *S_Y,c_*, and *S_X,c_* , were based on a conditional autoregressive (CAR) model. A directed acyclic graph (DAG) for the overall model is illustrated in Figure 2. Note that observed quantities are denoted as squares and unobserved quantities are denoted as circles.

### Spatial error structure and Bayesian estimation procedures

The spatial error terms *s_Y,ci_* and *s_X,ci_* are assumed to follow a spatial distribution defined by the CAR model (Besag et al. 1991). If we let *S__i_* denote the vector of spatial residual errors, excluding the subject *i*, the CAR model specifies that,


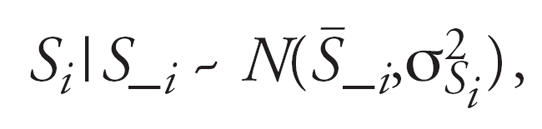


where


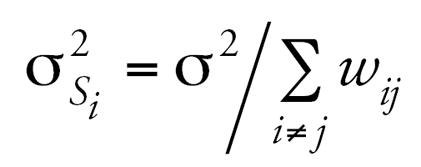


and


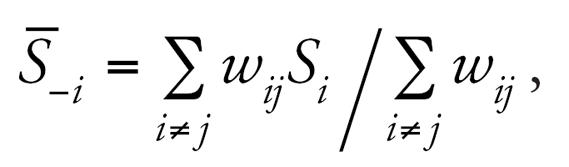


based on a weight matrix, *W_N_*_×_ *_N_* = [*w_ij_*]*_N_*_×_ *_N_*, specified to determine the amount of spatial similarity between all pairs of individuals, *i,j*. A first approximation for this weight matrix is to set *w_ij_* = 1 if areas *i* and *j* are “adjacent” to one another and zero otherwise. This is the kind of similarity matrix used to define all within-community spatial error terms, namely, *s_Y,ci_* and *s_X,ci_*. To construct these adjacency-based similarity matrices, ArcGIS 9.0/ArcMap 9.1 software packages (ESRI, Redlands, CA) were used to produce the Thiessen polygons for each subject where each polygon contains exactly one individual. Thiessen (sometimes called “Voronoi’) polygons are defined by a set of “center” points where each polygon is defined as the set of all points that are closer to a particular center than any other center. Using these polygons, adjacency-based weight matrices were constructed.

Thiessen polygons were used as a first approximation of possible spatial autocorrelation in health and environmental data. Because there is little prior evidence available on the likely spatial associations among subjects, the first-order connectivity matrix based on nearest neighbor proximity is used. This is a common approach in studies when little is known about the spatial processes that generate similarity of attributes by proximity (Odland 1988). The model is capable of adjustments for more informed spatial matrices when prior information is available, such as likely walking distances for the children.

The between-community spatial residual error terms *S_Y,c_* and *S_X,c_* were assumed to follow a CAR model with elements of the weight matrix specified as the inverse of driving distance between two communities. Because the subjects in this study were living in separate, disjoint communities all within a relatively small area within Southern California (an area of about 500 km at its maximum distance), most subjects would travel from one community to another via automobile. Therefore, community-level spatial correlation is reasonably well estimated by the driving distance between the communities. These driving distances were obtained by taking the average distances to drive in both directions for each pair of communities. Each one-way driving distance was obtained from the online mapping site Mapquest (2006). This community-level residual error leads to robust estimates of spatial errors (Borgoni et al. 2003).

The main structure of the Bayesian estimation procedures was described previously (Molitor et al. 2006). Briefly, the Markov chain Monte Carlo (MCMC) method Gibbs sampling was used to estimate the parameters of our model using the WinBUGS software package (version 1.4.1; Spiegelhalter et al. 2003). The Bayesian models were run for 20,000 burn-in iterations followed by 100,000 iterations that were stored for computing posterior distributions of parameters of interest. (This program is available upon request from the first author of this article.) Diffuse priors were used on all parameters. The regression parameters were assigned *N*(0, τ*_N_*) priors, where τ*_N_* denotes precision with τ*_N_* = 10^−4^. All standard deviation parameters were given flat uniform priors, *U*(0,τ*_U_*) with τ*_U_* = 10. Throughout the analyses, all measures of NO_2_, both estimated and observed, distance to nearest freeway, and the predicted NO_2_ based on CALINE4, as well as the outcome, *Y_ci_*, were measured on a log scale. The log transformation of the lung function outcome helps satisfy the normality assumptions of the model as was established in previous analysis of CHS data (e.g., Gauderman 2004). The additional log transformation of the exposure variables allows parameter estimates to be interpreted as rates of change based on the concept of elasticity. The coefficient in front of a particular covariate is interpreted as the percent change in the response *Y*, corresponding to a 1% change in the value of the covariate *X*, assuming everything else in the model is held constant, which is established in the econometric regression literature (Gujarati 1995).

### Model comparisons

Several different models were fit to the data to examine the effects of including various amounts of spatial information into exposure model (Equation 2). The “base” model did not include any traffic-level exposure variables. In other words, *W_ci_* was removed from the exposure model (Equation 2), resulting in a new exposure model in which a random town-level intercept term is the only nonresidual term used to predict long-term NO_2_. Subsequent models were formed by including combinations of relevant traffic-related parameters; namely, models were formed by including/excluding various combinations of covariates in the term *W_ci_*. All these models were fit with and without the presence of spatial error terms in order to examine the usefulness of various traffic-related covariates in explaining the extent to which the relationship of interest (lung function and NO_2_) varied spatially.

For each model, we calculated the deviance information criterion (DIC) (Spiegelhalter et al. 2002), which can be viewed as a Bayesian analogue of the Akaike Information Criterion (AIC; Akaike 1973). This measure of model fit can be easily computed in WinBugs (Spiegelhalter et al. 2003), and it provides another way of comparing different modeling approaches.

## Results

Table 1 shows the results of the integrated Bayesian model without the spatial autoregressive terms included; the bottom part shows results obtained with the spatial error terms. Comparison with previous results without spatial error allows for explicit testing of the contribution that spatial error makes to refining exposure–response relationships. Table 1 also gives DIC values computed using different models, with smaller values indicating a better model fit. Smaller DIC values were associated with models that resulted in tighter posterior credible intervals for the parameters of interest.

**Figure 3:**
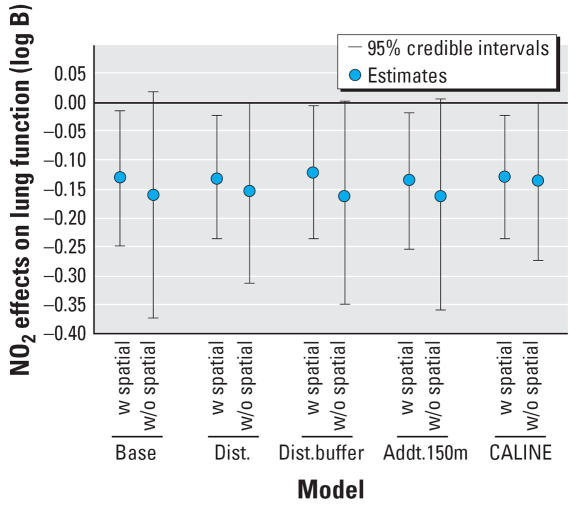
Spatial versus nonspatial effects across models. Abbreviations: w, with; w/o, without.

**Figure 4:**
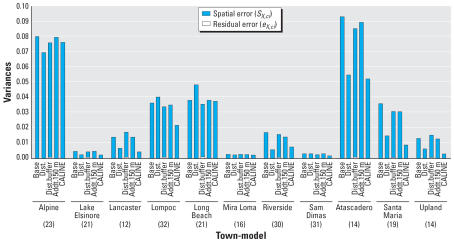
Variances of the individual-level spatial and independent residual terms for each California community in the exposure model Equation 2 for different choices of exposure predictors. (See text for definition of the variances plotted.) Numbers in parentheses indicate sample size.

**Figure 5:**
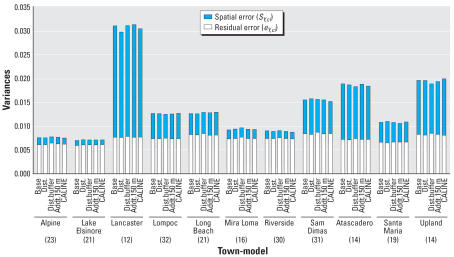
Variances of the individual-level spatial and independent residual terms for each community in the lung function model Equation 1 for different choices of exposure predictors. (See text for definition of the variances plotted.)

**Figure 6:**
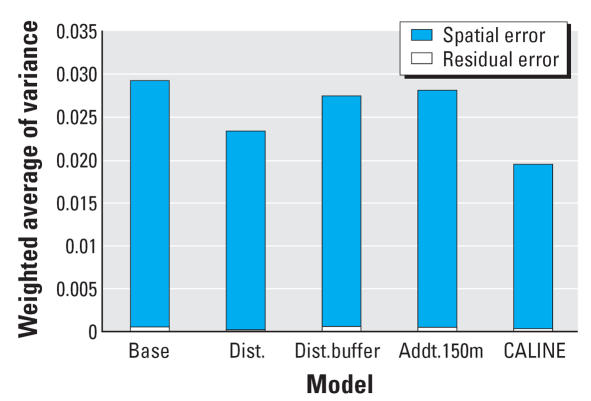
Variances of the community-level spatial and independent residual terms in the exposure model Equation 5 for different choices of exposure predictors. (See text for definition of the variances plotted.)

**Figure 7:**
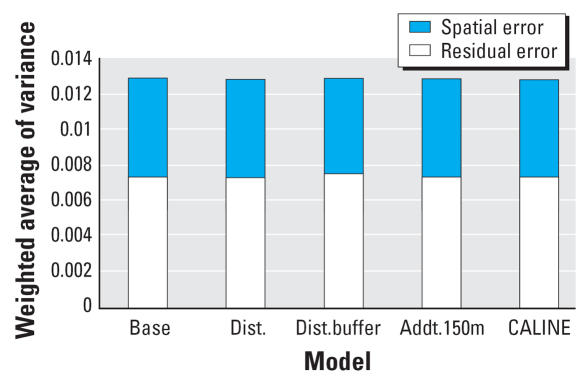
Variances of the community-level spatial and independent residual terms in the lung function model Equation 4 for different choices of exposure predictors. (See text for definition of the variances plotted.)

All models show a negative association between lung function and long-term exposure to NO_2_, meaning that higher air pollution exposure is associated with decreased lung function as measured by FVC. Models may be interpreted as log–log elasticities, such that a value of −0.14 means that for every 10% increase in long-term NO_2_ exposure, there is a decrease of 1.4% in lung function. The posterior 95% credibility intervals for the effect of NO_2_ on lung function are consistently narrower in models that use spatial residual terms compared with models without spatial errors included. The point estimates are also consistently smaller in the spatial models. Figure 3 graphically displays the increase in parameter estimate precision obtained when spatial information is included in the modeling process. As expected, estimates from the base model, namely, the model with no traffic related covariates, were changed the most by the inclusion of spatial information in estimating the residual errors. Table 1 also shows that the model with the narrowest credible interval for the effect of air pollution on lung function is the model that includes spatial errors and the CALINE4 dispersion model estimates. In contrast to the base model, the CALINE4 model includes the most exposure information, and as expected, this model is least affected by inclusion of the spatial error term. Figures 4 and 5 display the variances of the individual-level spatial and independent residuals for each community for the exposure and lung function models, respectively. Figures 6 and 7 show the corresponding variances of the community-level spatial and independent residual error terms. The within-community variances of the individual-level spatial residual terms are computed at each iteration of the Gibbs sampler to be


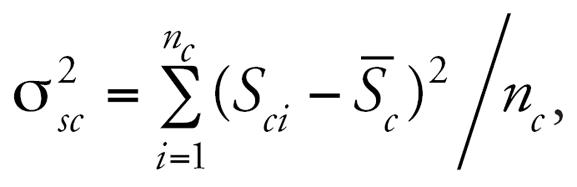


and these are then averaged across Gibbs samples; the variances of the independent errors are computed similarly with *e_ci_* replacing *S_ci_* . The variances of the community-level spatial and independent error terms across all subjects are defined to be the average across Gibbs samples of the within-community variances, namely,


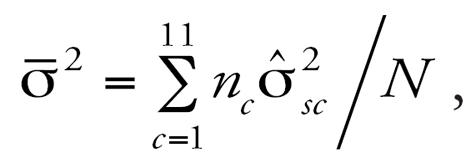


where


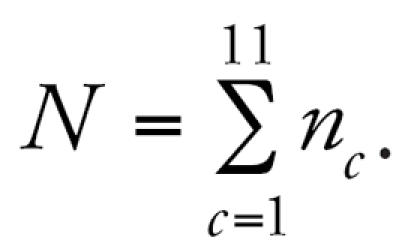


Posterior distributions are obtained for each of these community-specific parameters, and from these posterior means, each


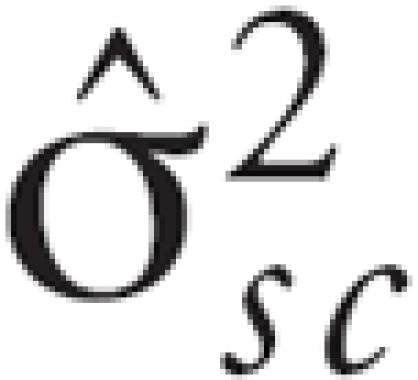


is obtained. It is evident from these figures that the spatial error terms were of much greater value in estimating long-term NO_2_ exposure than in modeling lung function. We have not reported results from the between-community spatial variances because these were very small.

**Figure 8:**
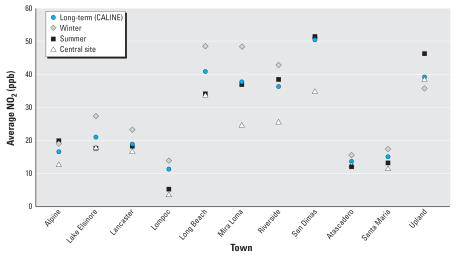
Comparison of different levels of NO_2_.

Figure 8 graphically compares average modeled estimates of long-term NO_2_ with observed seasonal and central-site averages. Although this figure displays only posterior averages of modeled exposure, the MCMC framework fully incorporates the uncertainty in these modeled estimates in the estimation of all model parameters.

## Discussion and Conclusion

Recent interest in health effects of air pollution requires a better understanding of which exposure models should be used in epidemiologic investigations. Our results are consistent with previous work in the entire CHS cohort demonstrating associations between lung function and NO_2_ measurements made at community central site monitors and between lung function and local variation in traffic exposure (Gauderman et al. 2007). A few European studies have examined associations of childhood lung function and local variation within communities of exposure indicators to traffic-related pollutants, with inconsistent results (Brunekreef et al. 1997; Janssen et al. 2003; Sugiri et al. 2006; Wjst et al. 1993).

The results presented in this article extend previous methodologic work (Molitor et al. 2006) by improving exposure assessment through the consideration of spatial correlation in air quality. In this previous work, we reported that multilevel Bayesian models without spatial errors performed better than simpler, one-level frequentist-based approaches (Molitor et al. 2006). The models with spatial error structures that have been proposed here represent a further improvement in modeling these data, as demonstrated in Table 1.

Our analysis reveals a range of point estimates and credible intervals, depending on which predictors were considered and whether spatial error terms are included. In the base model with only central site data, we obtained the widest credible intervals and large point estimates (in absolute value). Comparing the results in Table 1 without spatial errors for the base model and the model with the smallest credibility interval, the CALINE4 model, the point estimate is nearly 18% greater (in absolute value) for the base model and the credible interval is more than 45% wider for the base model when compared with that obtained from the CALINE4 model. A similar comparison between the CALINE4 model and the distance model shows a point estimate increase (in absolute value) of about 13% and a credible interval width increase of more than 14%. In both cases, exclusion of the more refined exposure information appears to inflate both the point estimate (in absolute value) and the uncertainty of that estimate. This situation differs from a standard regression setting, where one may compare the ability of a single covariate to predict an observed outcome with a model consisting of several covariates used to predict the same observed outcome. Here, observed covariate information such as traffic-related covariates and observed seasonal NO_2_ levels are not directly used to predict levels of lung function. Rather, these observed quantities are combined to estimate an unobserved latent variable, namely, long-term NO_2_ exposure, and this unobserved NO_2_ exposure is then used to predict the observed outcome—lung function. In this setting, with the data available, models with informative covariates and informative spatial error terms provide slightly smaller estimates of the effect of long-term NO_2_ on lung function, with tighter confidence levels. The estimates of health effects in this sample are sensitive to the exposure models used for analysis. Models with less robust information, such as the distance metric, tend to inflate both point estimates and statistical uncertainty, at least in the latent variable setup used with the current data. Further research with simulations and other health data sets is needed before drawing definitive conclusions about the best exposure metrics.

Comparison of Figure 6 with Figure 7 illustrates how the exposure and health models differ. Health models tend to have lower variance overall, and for the most part they are dominated by nonspatial residual error. Exposure models, in contrast, are dominated by spatial error, and they have higher variances overall. This is not surprising, given that it is likely that spatial heterogeneity of genetic and other factors such as diet may contribute to lung function, whereas NO_2_ pollution is caused by near-source traffic emissions or consistent transport from neighboring communities.

In the health-plus-exposure models, there is heterogeneity in the residual variance between the communities. For example, in the health model, the communities of Lancaster, Atascadero, and Upland have the largest unexplained variance. These communities are in different locations some hundreds of kilometers apart. Thus there is no obvious underlying similarity or spatial pattern in how community location and characteristics influence the residual variation in lung function.

In contrast, the exposure models perform much better in the inland areas of the Los Angeles Basin with respect to the magnitude of residual errors displayed in Figure 4. With the exception of Long Beach (a coastal community), most of the predictions in the Basin appear superior to those outside the Basin. Atascadero is poorly predicted by the model as are Lompoc, Santa Maria, and Alpine, all outside the basin. This may be because of the relatively lower levels of NO_2_ in these locations and the associated lower range of exposure.

Regarding spatial errors, one could use a Bayesian geostatistical kriging model of the form described in Diggle et al. (1998) as opposed to the CAR model used in the present article. The Bayesian kriging model assumes that spatial errors are modeled using a multivariate Gaussian distribution with covariance matrix expressed as a parametric function of the distance between pairs of points. This model is useful if one is primarily interested in making predictions of exposure on the spatial surface. For example, one may be interested in predicting levels of NO_2_ exposure at homes not measured in the pilot study. To facilitate the prediction of exposure, this model assumes stationarity, in that the amount of spatial correlation between two points is simply a function of the Euclidian distance between the points. Because we are primarily interested in assessing the effect of exposure on lung function and not in spatial prediction, and because assumptions of stationarity would questionable in our context, we have decided against using this model here.

Through examination of DIC, spatial autocorrelation in the outcome and exposure, and the subsequent impacts on point estimates and credible intervals, we have developed a framework for assessing spatial exposure model performance. In most cases, we were able to improve the certainty of our health effects estimates with information on residual spatial autocorrelation, but these improvements were, as expected, more pronounced in models that contained less informative exposure information. Exposure models with small (good) DIC had relatively less improvement from additional spatial information. This finding suggests a more general approach for assessing model performance where the point estimates and confidence intervals are more robust to inclusion of additional information, probably because of less bias in the initial estimates from nonindependence in the observations, particularly from excluded exposure information. As noted below, the generalizability of these findings is limited by the sample size used, but this will be partly addressed in future research.

There are limitations to this study that merit attention in future research. We have exposure information from only two 2-week periods in different seasons measured at the home. Although there are more field measurements than in most similar large epidemiologic investigations, it is possible that our estimates are not an accurate depiction of long-term exposure because of temporal variation in exposure. However, the measurement model (Equation 3) is not written in the way classic measurement error models are generally written, where observed measures of exposure are assumed to deviate around true unobserved exposure values with zero-error residuals. Instead, we have incorporated an extra term that calibrates local measurements for temporal variation as assessed by the central site measurements.

Furthermore, the relatively small sample size, although drawn from a larger cohort, may not be representative of the general population or of the exposure experienced by the entire cohort. Other analyses suggested few significant differences between this sample and the larger cohort (Gauderman et al. 2005), but caution must be exercised in comparing these results to those of the full cohort (i.e., Gauderman et al. 2007).

We have collected subsequent information from over 1,000 locations in a related study over three seasons that will allow us to address the weaknesses described previously. Also, our unified modeling framework will allow us to combine information from the entire cohort, as individual-level exposures that may not exist in the larger cohort study but are present in the pilot study can be imputed in a way that fully utilizes all available covariate information. Because of the small sample within each community in the pilot study analyzed for this article, we were unable to evaluate other predictors of exposure based on other land uses (Jerrett et al. 2005a), a method that has been used in a few health studies (Brauer et al. 2002) and has performed as well or better than dispersion models like CALINE4 when predicting exposures at unmeasured locations (Briggs et al. 2000). We will address this limitation as well in future studies with the larger samples of measured exposures.

Here we sought to examine how different models of intraurban air pollution exposure classify and predict FVC in an integrated Bayesian modeling framework. Building on the CHS (Gauderman et al. 2004, 2007) and related methodologic developments (Molitor et al. 2006), we assessed three intraurban predictors (i.e., distance to a freeway, traffic density, and CALINE4 dispersion models) in a Bayesian measurement error framework. Traffic density and distance buffer are commonly used in epidemiologic studies (Jerrett et al. 2005b), and CALINE has been used in a few studies (e.g., Gauderman et al. 2005, 2007; McConnell et al. 2006). The novelty to our method is the inclusion of between- and within-community spatial autocorrelation terms and the systematic testing of different exposure models. Results obtained through the Bayesian framework suggest that the inclusion of residual spatial terms can reduce uncertainty in the prediction of exposures and associated health effects. The findings also imply that more informative exposure models appear to reduce uncertainty in health effects estimation.

## Figures and Tables

**Table 1:** NO_2_ effects on lung function.

Model	Estimate (% change)	95% credible interval	Width of 95% CI	Total DIC (small is better)
Without spatial errors				
Base	−0.159	−0.374 to 0.017	0.390	−308.560
Base + dist	−0.152	−0.312 to −0.005	0.308	−331.160
Base + dist.buffer	−0.163	−0.348 to 0.002	0.350	−311.526
Base + addt150m	−0.163	−0.360 to 0.005	0.365	−311.046
Base + caline	−0.135	−0.273 to −0.004	0.269	−349.570
With spatial errors				
Base	−0.131	−0.249 to −0.015	0.234	−368.481
Base + dist	−0.131	−0.238 to −0.023	0.215	−408.516
Base + dist.buffer	−0.122	−0.238 to −0.005	0.233	−365.369
Base + addt150m	−0.135	−0.252 to −0.020	0.233	−370.630
Base + caline	−0.129	−0.236 to −0.023	0.213	−418.446

dist, distance to nearest freeway. Base = NO_2_ exposure level was estimated without any predictors of exposure level (level 2 in Equation 2). Base + dist = NO_2_ exposure level was estimated by distance to a freeway in exposure level (level 2 in Equation 2). Base + dist.buffer = NO_2_ exposure level was estimated by the categorized distance to a freeway in exposure level (level 2 in Equation 2). Base + addt150m = NO_2_ exposure level was estimated by the traffic counts within 150 m in exposure level (level 2 in Equation 2). Base + CALINE = NO_2_ exposure level was estimated by predicted NO_2_ level based on CALINE model.

## References

[b1-ehp0115-001147] Ackermann-Liebrich U, Leuenberger P, Schwartz J, Schindler C, Monn C, Bolognini G (1997). Lung function and long term exposure to air pollutants in Switzerland. Study on Air Pollution and Lung Diseases in Adults (SAPALDIA) Team. Am J Respir Crit Care Med.

[b2-ehp0115-001147] Akaike H, Petrov BN, Cs′aki F (1973). Information theory and an extension of the maximum likelihood principle. Proceedings of the Second International Symposium on Information Theory.

[b3-ehp0115-001147] Benson P (1984). CALINE4—a Dispersion Model for Predicting Air Pollution Concentration near Roadways.

[b4-ehp0115-001147] Besag J, York J, Mollie A (1991). Bayesian image restoration, with two applications in spatial statistics. Ann Inst Stat Math.

[b5-ehp0115-001147] Borgoni R, Billari FC (2003). Bayesian spatial analysis of demographic survey data. Demographic Res.

[b6-ehp0115-001147] Brauer M, Hoek G, Van Vliet P, Meliefste K, Fischer P, Gehring U (2003). Estimating long-term average particulate air pollution concentrations: application of traffic indicators and geographic information systems. Epidemiology.

[b7-ehp0115-001147] Brauer M, Hoek G, Van Vliet P, Meliefste K, Fischer P, Wijga A (2002). Air pollution from traffic and the development of respiratory infections and asthmatic and allergic symptoms in children. Am J Respir Crit Care Med.

[b8-ehp0115-001147] Briggs DJ, de Hoogh C, Gulliver J, Wills J, Elliott P, Kingham S (2000). A regression-based method for mapping traffic-related air pollution: application and testing in four contrasting urban environments. Sci Total Environ.

[b9-ehp0115-001147] Brunekreef B, Holgate S (2002). Air pollution and health. Lancet.

[b10-ehp0115-001147] Brunekreef B, Janssen NA, de Hartog J, Harssema H, Knape M, van Vliet P (1997). Air pollution from truck traffic and lung function in children living near motorways. Epidemiology.

[b11-ehp0115-001147] Chaix B, Gustafsson S, Jerrett M, Kristersson H, Lithman T, Boalt Å (2006). Children’s exposure to nitrogen dioxide in Sweden: investigating environmental injustice in an egalitarian country. J Epidemiol Community Health.

[b12-ehp0115-001147] Diggle PJ, Tawn JA, Moyeed RA (1998). Model-based geostatistics. Appl Stat.

[b13-ehp0115-001147] Gauderman WJ, Avol E, Gilliland F, Vora H, Thomas D, Berhane K (2004). The effect of air pollution on lung development from 10 to 18 years of age. N Engl J Med.

[b14-ehp0115-001147] Gauderman WJ, Avol E, Lurmann F, Kuenzli N, Gilliland F, Peters J (2005). Childhood asthma and exposure to traffic and nitrogen dioxide. Epidemiology.

[b15-ehp0115-001147] Gauderman WJ, Vora H, McConnell R, Berhane K, Gilliland F, Thomas D (2007). Effect of exposure to traffic on lung development from 10 to 18 years of age: a cohort study. Lancet.

[b16-ehp0115-001147] Gujarati D (1995). Basic Econometrics.

[b17-ehp0115-001147] Hewitt CN (1991). Spatial variations in nitrogen dioxide concentrations in an urban area. Atmos Environ.

[b18-ehp0115-001147] Hoek G, Brunekreef B, Goldbohm S, Fischer P, van den Brant P (2002). Association between mortality and indicators of traffic-related air pollution in the Netherlands: a cohort study. Lancet.

[b19-ehp0115-001147] Janssen NA, Brunekreef B, van Vliet P, Aarts F, Meliefste K, Harssema H (2003). The relationship between air pollution from heavy traffic and allergic sensitization, bronchial hyperresponsiveness, and respiratory symptoms in Dutch schoolchildren. Environ Health Perspect.

[b20-ehp0115-001147] Jerrett M, Burnett RT, Ma R, Pope CA, Krewski D, Newbold KB (2005a). Spatial analysis of air pollution and mortality in Los Angeles. Epidemiology.

[b21-ehp0115-001147] Jerrett M, Finkelstein M (2005b). Geographies of risk in studies linking chronic air pollution exposure to health outcomes. J Toxicol Environ Health.

[b22-ehp0115-001147] Mapquest (2006). Homepage.

[b23-ehp0115-001147] McConnell R, Berhane K, Yao L, Jerrett M, Lurmann F, Gilliland F (2006). Traffic, susceptibility, and childhood asthma. Environ Health Perspect.

[b24-ehp0115-001147] Molitor J, Molitor NT, Jerrett M, McConnell R, Gauderman J, Berhane K (2006). Bayesian modeling of air pollution health effects with missing exposure data. Am J Epidemiol.

[b25-ehp0115-001147] National Research Council (2002). Estimating the Public Health Benefits of Proposed Air Pollution Regulations.

[b26-ehp0115-001147] Odland J (1988). Spatial Autocorrelation.

[b27-ehp0115-001147] Peters JM, Avol E, Gauderman J, Linn WS, Navidi W, London SJ (1999a). A study of twelve Southern California communities with differing levels and types of air pollution. II. Effects on pulmonary function. Am J Respir Crit Care Med.

[b28-ehp0115-001147] Peters JM, Avol E, Navidi W, London SJ, Gauderman WJ, Lurmann F (1999b). A study of twelve Southern California communities with differing levels and types of air pollution. I. Prevalence of respiratory morbidity. Am J Respir Crit Care Med.

[b29-ehp0115-001147] Spiegelhalter DJ, Best NG, Carlin BP, van der Linde A (2002). Bayesian measures of model complexity and fit. J Roy Statist Soc B.

[b30-ehp0115-001147] Spiegelhalter D, Thomas A, Best N (2003). WinBUGS, Version 1.4 User Manual.

[b31-ehp0115-001147] Sugiri D, Ranft U, Schikowski T, Kramer U (2006). The influence of large-scale airborne particle decline and traffic-related exposure on children’s lung function. Environ Health Perspect.

[b32-ehp0115-001147] Wjst M, Reitmeir P, Dold S, Wulff A, Nicolai T, von Loeffelholz-Colberg EF (1993). Road traffic and adverse effects on respiratory health in children. BMJ.

[b33-ehp0115-001147] Zhu Y, Hinds W, Kim S, Shen S, Sioutas C (2002). Study on ultra-fine particules near a major highway with heavy-duty diesel traffic. Atmos Environ.

